# Models of deletion for visualizing bacterial variation: an application to tuberculosis spoligotypes

**DOI:** 10.1186/1471-2105-9-496

**Published:** 2008-11-27

**Authors:** Josephine F Reyes, Andrew R Francis, Mark M Tanaka

**Affiliations:** 1 School of Biotechnology and Biomolecular Sciences, University of New South Wales, Sydney 2052, Australia; 2 Evolution & Ecology Research Centre, University of New South Wales, Sydney 2052, Australia; 3 School of Computing and Mathematics, University of Western Sydney, South Penrith DC, NSW 1797, Australia

## Abstract

**Background:**

Molecular typing methods are commonly used to study genetic relationships among bacterial isolates. Many of these methods have become standardized and produce portable data. A popular approach for analyzing such data is to construct graphs, including phylogenies. Inferences from graph representations of data assist in understanding the patterns of transmission of bacterial pathogens, and basing these graph constructs on biological models of evolution of the molecular marker helps make these inferences. Spoligotyping is a widely used method for genotyping isolates of *Mycobacterium tuberculosis *that exploits polymorphism in the direct repeat region. Our goal was to examine a range of models describing the evolution of spoligotypes in order to develop a visualization method to represent likely relationships among *M. tuberculosis *isolates.

**Results:**

We found that inferred mutations of spoligotypes frequently involve the loss of a single or very few adjacent spacers. Using a second-order variant of Akaike's Information Criterion, we selected the Zipf model as the basis for resolving ambiguities in the ancestry of spoligotypes. We developed a method to construct graphs of spoligotypes (which we call spoligoforests). To demonstrate this method, we applied it to a tuberculosis data set from Cuba and compared the method to some existing methods.

**Conclusion:**

We propose a new approach in analyzing relationships of *M. tuberculosis *isolates using spoligotypes. The spoligoforest recovers a plausible history of transmission and mutation events based on the selected deletion model. The method may be suitable to study markers based on loci of similar structure from other bacteria. The groupings and relationships in the spoligoforest can be analyzed along with the clinical features of strains to provide an understanding of the evolution of spoligotypes.

## Background

The visualization of relationships among genotypes of bacterial isolates is a useful approach to addressing both evolutionary and epidemiological questions. Inferences from graph representations of data assist in understanding the patterns of transmission of bacterial pathogens. Presently, there are two approaches to visualization. The first class of methods is sequence-based, and these methods often produce phylogenetic trees, or dendrograms. These trees are used extensively to represent relatedness of isolates that have been identified by almost any typing procedure. However, the models of sequence evolution upon which phylogenetic methods depend are not appropriate for many markers that are not sequence-based. Also, because many of these markers evolve rapidly enough to generate intra-specific variation, it is preferable to show direct relationships between genotypes. Bacterial isolates often cluster into the same genotype, and dendrograms are not suited to showing these clusters.

The second class of methods produce "network-like" graphs that show direct relationships between clusters of genotypes. Some examples of this second class of methods are found in the works of Zhu et al. [1], Tanaka and Francis [2] and Excoffier and Smouse [3]. The genoclouds in Zhu et al. [1] consist of related isolates of *Neisseria meningitidis *that are grouped according to criteria that minimize genetic, temporal and physical distances. The result is a parsimonious tree that depicts the relationships between the genoclouds. Similarly, Tanaka and Francis [2] proposed cluster-graphs where isolates of *Mycobacterium tuberculosis *sharing the same genotype are assigned into clusters, and all possible close relationships between these clusters are shown. In both these methods, clusters are associated with epidemiologically linked cases of infection. Guernier et al. [4] developed a technique of representation that is based on the cluster-graph, with two additional elements included: (1) concentric circles to show the number of possible mutation steps between spoligotypes, and (2) hypothetical intermediate nodes to visualize possible links between clades of spoligotypes known to be related. Excoffier and Smouse [3] used an analysis of molecular variance to construct minimum spanning trees and networks to represent genetic relatedness. See Posada et al. [5] for a more general discussion of graphical methods to represent relationships.

The eBurst package [6,7] is designed for visualizing data from multi-locus sequence typing (MLST). Isolates that have similar sequence types are assigned to disjoint groups, where similarity depends on the number of shared alleles in the MLST profile. The radial layout of eBurst diagrams shows groups or subgroups of related genotypes, centered around the inferred founding genotypes. The complexity of an eBurst diagram suggests the age of the clonal complex; a clonal complex is considered young when its structure is simple and older when its structure is complex. There are many genotyping technologies enabling the study of genetic variation in bacteria. Here, we focus on spacer oligonucleotide typing (spoligotyping), a technique that exploits polymorphism in the direct repeat (DR) region of *M. tuberculosis *[8,9]. This method has gained widespread use for differentiating isolates of *M. tuberculosis *over the last decade [10]. The DR region is composed of numerous identical 36-base-pair direct repeats, interspersed by nonrepetitive short sequences or direct variable repeats (DVRs) called spacers. Mechanisms known to cause variation in this locus are homologous recombination between adjacent or spatially distant DRs leading to deletion, and transposition and recombination of IS*6110 *elements in the DR locus [9,11].

The DR locus has been identified as a hotspot for the integration of insertion elements in the chromosome of *M. tuberculosis *complex strains [12]. Such insertion into a spacer sequence can lead to the apparent deletion of that spacer [13]. It is presumed that spacers cannot be recovered when lost, since there is little or no recombination observed between strains [14].

It is possible that there is a relationship between the presence of some number of spacers in specific positions and the transmission rate of a strain, as seen by the lack of a length of spacers in the W-Beijing strain, which is prevalent in many data sets. However, in our model, we assume that a deletion event has no relation with the transmission rate, and any such relationship is beyond the scope of our study. We refer the reader to papers that discuss the importance of studying the W-Beijing type and its transmission [15,16].

In this paper we examine the problem of determining a plausible evolutionary history of a sample of tuberculosis spoligotypes using an explicit model of the evolution of the DR locus. We start with the cluster-graph construct of Tanaka and Francis [2] to represent all possible mutation events in a sample of spoligotypes. Nodes of a cluster-graph represent distinct spoligotypes in a sample, and edges drawn between nodes determine the possible mutation events. By mutation we mean a deletion of one or more spacers in a spoligotype. Even for moderately large samples, this can lead to a tangled network of relationships between spoligotypes, which can hinder further analysis. In particular, many spoligotypes appear to have originated from multiple parent spoligotypes. One solution to this problem is to randomly sample edges from a set of multiple inbound edges [17]. However, some edges (mutation events) may be more likely than others to explain the origin of a given spoligotype. We formulate a variety of models to describe the deletion processes that generate variation in the DR locus, and identify an appropriate model using Akaike's Information Criterion. The selected model can then be used to choose a single inbound edge into a specific spoligotype. Applying this procedure to each spoligotype with multiple inbound edges in a sample, we can refine the cluster-graph. We call the resulting graph a spoligoforest.

## Methods

We present several candidate deletion models of spoligotype evolution, then compare them using a second-order form of the Akaike's Information Criterion (*AIC*_*c*_) and data from selected published spoligotype samples (see Table 1). In this section we begin with the underlying assumptions about spoligotypes and their evolution. We then outline the procedure for model selection and finally describe the models.

**Table 1:** Spoligotype data sets used in this analysis

Publication	Isolates^*a*^	Spoligotypes	Location
Soini et al. [35]	1283	227	USA
David et al. [36]	665	159	Portugal
Jou et al. [37]	420	113	Taiwan
Sajduda et al. [38]	251	91	Poland
Nikolayevskyy et al. [39]	225	73	Ukraine
Easterbrook et al. [40]	224	79	Zimbabwe
Mokrousov et al. [41]	123	14	China
Godreuil et al. [42]	120	39	Burkina Faso
Toungoussova et al. [43]	114	17	Russia
Sola et al. [44]	104	56	Italy
Millet et al. [45]	100	21	Japan
Sun et al. [46]	68	41	Singapore
Pfyffer et al. [24]	65	13	Azerbaijan
Banu et al. [47]	48	18	Bangladesh
Douglas et al. [48]	11	8	Philippines

### Assumptions on the evolution of spoligotypes

A spoligotype consists of 43 binary characters. Each binary character denotes the presence or absence of a spacer in the DR locus of *M. tuberculosis*. The copy number of a spoligotype refers to the number of spacers present in its binary pattern. It is assumed that a mutation event involves the deletion of any number of adjacent spacers from the spoligotype; deleted spacers are not recovered, so that the spoligotype resulting from a mutation always contains fewer spacers than the parent. For our purposes we regard the different mechanisms that influence variation in the DR locus to be indistinguishable. Furthermore, deletion is the only source of variation. In our model we assume that a deletion event has no relation with the transmission rate. We assume that the mutation rate is low enough relative to the transmission rate that infected individuals carry only a single strain of *M. tuberculosis *with a specific spoligotype. When this infecting strain mutates, it is replaced by a strain with a different spoligotype that has not been observed elsewhere in the sample. Consequently, in any sample, a given spoligotype can have at most one possible parent spoligotype, but potentially many descendants.

### Data sets and cluster-graphs

Given that spoligotypes mutate by deletions of adjacent blocks of spacers, we would like to know whether some lengths of deleted adjacent blocks are more probable than others. Specifically, we would like to find the frequency of each deletion size. There are many published tuberculosis data sets using spoligotyping as a marker containing the information required for our purposes. We selected fifteen published data sets that provide the spoligotyping pattern of each *M. tuberculosis *isolate in the sample and the number of isolates that cluster into each pattern (see Table 1). We consider that individuals within a sample are sufficiently close to each other for transmission to occur. These data sets come from various parts of the world, and vary in statistical features such as *RTI*_*n*-1 _(in the range (0.3279,0.8055)), number of singletons (7,105), average cluster size (1.8,6.7) and *θ*-estimate (2.73,66.25). Some of these quantities are discussed in [2] and [10].

We use cluster-graphs as described in Tanaka and Francis [2]. We group isolates that have the same spoligotype into clusters; each cluster is drawn as a node, and a possible single-event deletion that relates two clusters (spoligotypes) is represented by a directed edge. Possible deletion events are established by pairwise comparisons of spoligotype patterns. We define a *spoligoforest *to be a cluster-graph in which a single parent is chosen for each cluster having one or more parents. Some clusters in a cluster-graph already have a unique parent, and are likely to represent true deletions. This set of unambiguous deletion events forms the sample of deletion lengths for the model selection. Table 2 (column 4) shows the number of such edges from each data set. We assume that mutations occur independently of the state of the population, and hence edges, which represent mutations, are independent. The edges from the different data sets, representing independent deletion events, are pooled together in order to analyze the frequency of deletion lengths. We obtain an empirical distribution of deletion lengths represented by the unambiguous edges from the fifteen data sets. The total pool of analyzed unambiguous edges consisted of 339 deletion events.

**Table 2:** Data sets and their graph features

Published data set (First Author)	Cluster-graph edges^*a*^	Spoligoforest edges ^*b*^	Unambiguous edges^*c*^
Soini	445	126	56
David	403	129	60
Jou	366	84	45
Sajduda	342	63	28
Nikolayevskyy	212	59	29
Sola	137	45	22
Easterbrook	90	53	32
Sun	42	20	15
Godreuil	28	22	18
Mokrousov	27	10	6
Millet	22	13	7
Toungoussova	22	11	8
Banu	13	8	6
Douglas	11	6	3
Pfyffer	8	5	4

Pooled edges			339

^*a *^Number of single-event deletions in the cluster-graph.

^*b *^Number of single-event deletions in any spoligoforest derived from the data.

^*c *^Number of nodes in the cluster-graph having a single parent node.

### Candidate models for spacer deletion length in pattern mutations

Our goal is to find a model that best describes the underlying process generating the distribution of lengths of spacer deletions in the inferred mutation events. We formulated several candidate models based on standard discrete distributions and various hypotheses on spacer deletion lengths. For each model we found the maximum likelihood estimators (MLEs) of the parameters, analytically when possible, and numerically otherwise. Let the observed number of deletions of length *i *be *x*_*i*_, where *i *can take values from 1 to 43, let *m *be the total number of mutations analyzed (the unambiguous edges, in this case 339), and let $\bar{x}$ be the mean deletion length. Let the random variable *K *describe the deletion length associated with a mutation event. In each of the candidate models, let *P*(*K *= *k*) (or *P*(*k*)) be the probability mass function. The corresponding likelihood function is $ℒ(p|x)=\prod_{k=1}^{\infty }{\left[P(k)\right]}^{x_{k}}$, where **p **is the vector of parameters, *x *is the frequency of deletion lengths collected from the data sets, *k *is the deletion length, and *x*_*k *_is the frequency of the class of deletions with length *k*.

For each of the models, we computed the value of the second-order variant of Akaike's Information Criterion (*AIC*_*c*_) [18] to select a parsimonious model. The *AIC*_*c *_is given by the formula

(1) $$AIC_{c}=-2\ln ℒ(\hat{p}|x)+\frac{2ms}{m-s-1}$$

where ℒ is the likelihood, $\hat{p}$ is the vector of parameters at the maximum likelihood, *x *is the frequency of deletion lengths collected from the data sets, *m *is the sample size (the number of edges) and *s *is the number of parameters in the model. Models with low relative *AIC*_*c *_values are favoured. A summary of the probability mass functions and (MLEs) can be found in Table 3.

**Table 3:** Probability mass functions and maximum likelihood estimators of the models considered

Model name	support	Probability mass function	Maximum likelihood estimator
Geometric	*k *∈ [1, ∞)	*P*(*K *= *k*) = *P*(*k*) = *p*^*k*-1^(1 - *p*)	$\hat{p}=1-\frac{1}{\bar{x}}$

Negative binomial	*k *∈ [1, ∞)	$P(k)=\frac{{\left(1-p\right)}^{r}}{1-{\left(1-p\right)}^{r}}\left(\begin{array}{c} k+r-1\\ r-1 \end{array}\right)p^{k}$ where *k, r *≥ 1	$\hat{p}=1-\frac{\hat{r}}{\hat{x}}$

Conditional Poisson	*k *∈ [1, ∞)	$P(k)=e^{-\lambda }\frac{\lambda^{k}}{k!(1-e^{-\lambda })}$ where *k *≥ 1, *λ *> 0	Solution to $\bar{x}=\hat{\lambda }/(1-e^{-\hat{\lambda }})$

Logarithmic series	*k *∈ [1, ∞)	$P(k)=-\frac{\theta^{k}}{k\log(1-\theta )}$	Solution to $\bar{x}=\frac{-\hat{\theta }}{\left(1-\hat{\theta })\log(1-\hat{\theta }\right)}$

Zeta	*k *∈ [1, ∞)	$P(k)=\frac{k^{-\rho }}{\sum_{d=1}^{\infty }d^{-\rho }}$ where *ρ *> 1	Estimated numerically

Zipf	*k *∈ [1, 43]	$P(k)=\frac{k^{-\rho }}{\sum_{d=1}^{43}d^{-\rho }}$ where *ρ *> 1	Estimated numerically

Uniform	*k *∈ [1, 43]	$P(k)=\frac{1}{43}$	$\prod_{k=1}^{43}{\left(\frac{1}{a}\right)}^{x_{k}}$

Uniform	*k *∈ [1, *a*]	$P(k)=\frac{1}{a}$ where 1 ≤ *a *≤ 43	Estimated numerically

Empirical	*k *∈ [1, 43]	$P(k)=\frac{x_{k}}{m}$	$\prod_{k=1}^{43}P{\left(k\right)}^{x_{k}}$

#### Geometric model

Consider a deletion mechanism that moves along the DR region, making independent attempts to remove a spacer. Let *k *be the number of spacers this mechanism is able to remove, and the constant probability of the removal of a spacer is *p*. The probability mass function is *P*(*k*) = *p*^*k*-1^(1 - *p*), where *k *≥ 1 and 0 <*p *< 1. The MLE for *p *is $\hat{p}$ = 1 -1/$\bar{x}$.

#### Negative binomial model

To generalize the geometric model, we define the negative binomial parameters *p *and *r*. Consider a deletion mechanism that involves *r *rounds of spacer deletion, so that in total *k *spacers are removed. Each of these *r *rounds removes a geometrically distributed number of spacers with parameter *p*. The probability mass function is

(2) $$P(k)=\frac{{\left(1-p\right)}^{r}}{1-{\left(1-p\right)}^{r}}\left(\begin{array}{c} k+r-1\\ r-1 \end{array}\right)p^{k}$$

where *k, r *≥ 1 and 0 <*p *< 1. The MLEs were found by solving $\hat{p}$= 1 - $\hat{r}$/$\bar{x}$ for $\hat{r}$ and $\hat{p}$. This was done by considering the equations conditionally on 1 ≤ $\hat{r}$ ≤ 10 to solve for the values of $\hat{p}$ and inspecting the likelihood.

#### Conditional Poisson model

In this model, the deletion process results in the loss of *k *spacers distributed as a Poisson parameter *λ *> 0, conditional on *k *≥ 1. The probability mass function is

(3) $$P(k)=e^{-\lambda }\frac{\lambda^{k}}{k!(1-e^{-\lambda })}$$

with MLE $\hat{\lambda }$ such that $\bar{x}=\hat{\lambda }/(1-e^{-\hat{\lambda }})$.

#### Logarithmic model

Suppose the deletion process causes the loss of *k *spacers, following a logarithmic distribution, given by the probability mass function

(4) $$P(k)=-\frac{\theta^{k}}{k\log(1-\theta )},$$

with 0 <*θ *< 1 and *k *≥ 1. The MLE is found by solving the equation $\bar{x}=\frac{-\hat{\theta }}{\left(1-\hat{\theta })\log(1-\hat{\theta }\right)}$ for $\hat{\theta }$.

#### Zeta model

Suppose the deletion process results in the loss of *k *spacers distributed as a zeta parameter *ρ*. The probability mass function is

(5) $$P(k)=\frac{k^{-\rho }}{\sum_{d=1}^{\infty }d^{-\rho }}$$

with *ρ *> 1 and *k *≥ 1. The MLE $\hat{\rho }$ can be calculated numerically.

#### Zipf model

If we restrict the loss of *k *spacers to the interval 1 ≤ *k *≤ 43, then the probability mass function for the zeta model can be written with a finite sum in its denominator, i.e.,

(6) $$P(k)=\frac{k^{-p}}{\sum_{d=1}^{43}d^{-p}}.$$

The MLE $\hat{p}$ can be found numerically.

#### Uniform model

We consider a deletion process that cuts spacers in lengths distributed uniformly across deletion lengths *k *up to some endpoint *a*. The probability mass function is *P*(*k*) = 1/*a *where *k *and *a *are integers such that 1 ≤ *k *≤ *a *and 1 ≤ *a *≤ 43. We denote this model as Uniform (V), where the endpoint *a *is a parameter, and is allowed to vary. The MLE $\hat{a}$ was found numerically. We also included a variation of the uniform model where *P*(*k*) = *p *for all values of 1 ≤ *k *≤ 43, with MLE $\hat{p}$. We refer to this model as Uniform (F), where the endpoint *a *is fixed at *a *= 43.

#### Empirical model

We include in the analysis a model that completely represents the empirical values of deletion frequencies in the fifteen data sets used as reference (see Table 1). The probability mass function is $P(k)=\frac{x_{k}}{m}$ where *x*_*k *_is the frequency of *k*-deletions and *m *is the total number of deletions, with 1 ≤ *k *≤ 43. Setting the parameters to be *p*_*k *_for each *k*, the values of the MLEs ${\hat{p}}_{k}$ are found analytically.

This model has 42 free parameters.

## Results

We begin with some general observations about the relative frequencies of different deletion lengths. We discuss the outcomes of the model selection procedure, and then apply the chosen model to a new visualization method for representing relationships of isolates of tuberculosis.

### Inferred pattern mutations of spoligotypes frequently involve short spacer deletions

**Figure 1:**
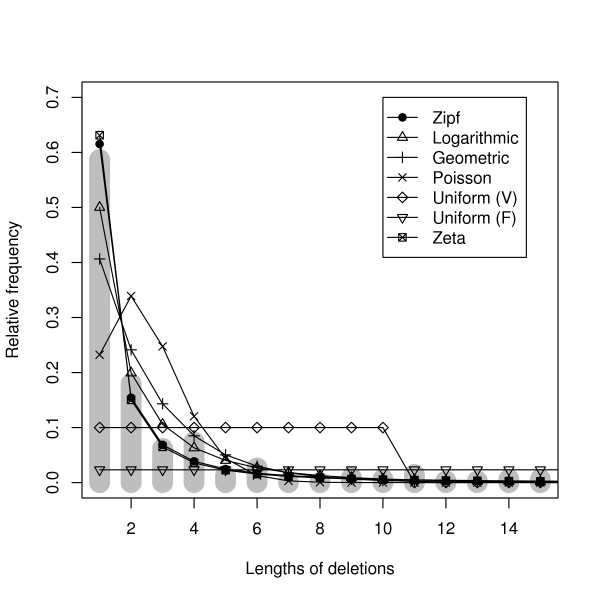
**Models**. Relative frequencies of the lengths of spacer deletions (shown here for deletion lengths 1 to 15) estimated by the models. The gray bars represent the empirical values. Uniform (V) refers to the uniform model with parameter $\hat{a}$ = 10, while Uniform (F) is the uniform model with $\hat{p}$ = 1/43.

The selected data sets independently show a high frequency of deletions of a single spacer. The pooled set of edges are shown in the gray bars of Figure 1. The 339 single-event deletions are distributed as shown in Table 4. Deletion lengths not appearing in Table 4 are not observed. The average number of spacers deleted is $\bar{x}$ = 2.46 and the standard deviation is 3.376. The skewed distribution of deletion sizes indicate a high number of short deletions, and very few longer deletions. Note, it is conceivable that spoligotypes that exist in the population but not sampled are intermediate in state between two sampled spoligotypes. If such spoligotypes exist and are sampled then the distribution would shift further towards shorter deletion lengths.

**Table 4:** Frequency of lengths of spacer deletions

Length	1	2	3	4	5	6	9	10	11	12	13	14	15	16	17	26	32
Frequency	199	61	21	25	4	9	3	2	5	1	1	2	2	1	1	1	1

### Model selection

We selected the model with the lowest value of *AIC*_*c *_(see Equation 1). This model is the Zipf model (Equation (6)). *AIC*_*c *_values and parameter estimates for some of the models are shown in Table 5. Figure 1 shows a plot of the relative frequencies for deletion lengths estimated by each of the models as well as the actual empirical values (gray bars). We verified that the selected model fits the individual data sets well by repeating the analysis separately for the individual data sets. The Zipf model often has the lowest *AIC*_*c*_, while the logarithmic series and geometric models are selected in some individual data sets (see Table 6).

**Table 5:** *AIC*_*c *_values of models with respect to pooled edges

Model name	No. of parameters	Parameter estimate	*AIC*_*c*_
Geometric^*a*^	1	$\hat{p}$ = 0.5935	1128.82
Negative binomial^*a*^	2	$\hat{r}$ = 1; $\hat{p}$ = 0.5935	1130.82
Conditional Poisson^*a*^	1	$\hat{\lambda }$ = 3.3697	1597.9
Logarithmic series^*a*^	1	$\hat{\theta }$ = 0.7967	1044.49
Zeta^*a*^	1	$\hat{\rho }$ = 2.0696	1033.46
Zipf *k *∈ [1, 43]	1	$\hat{p}$ = 1.9962	1025.23^*b*^
Uniform (V) *k *∈ [1, 10]	1	$\hat{a}$ = 10	1770.39
Uniform (F) *k *∈ [1, 43]	1	$\hat{p}$ = 1/43	2351.77
Empirical-based *k *∈ [1, 43]	42	${\hat{p}}_{k}$	1066.94

^*a*^Model has infinite support for *k*

^*b*^Selected model

**Table 6:** *AIC*_*c *_values of models in individual data sets for the best model and the Zipf model

Data set	Best model	Lowest *AIC*_*c*_	*AIC*_*c *_of Zipf
Soini	Log series	175.12	179.15
David	Zipf	155.58	155.58
Jou	Zipf	126.11	126.11
Sajduda	Zipf	93.60	93.60
Nikolayevskyy	Zipf	107.79	107.79
Sola	Zipf	55.80	55.80
Easterbrook	Log series	82.50	84.07
Sun	Zipf	21.64	21.64
Godreuil	Log series	60.60	62.84
Mokrousov	Zipf	20.17	20.17
Millet	Zipf	21.78	21.78
Toungoussova	Log series	34.47	35.23
Banu	Geometric	31.10	33.28
Douglas	Log series	19.83	20.05
Pfyffer	Geometric	22.86	23.70

### Visualizing relationships among spoligotypes

The selected model can now be used in a method to visualize relationships among *M. tuberculosis *isolates. For a specific data set consisting of *M. tuberculosis *isolates typed using spoligotyping, we represent each spoligotype by a node with area proportional to the number of isolates with that spoligotype pattern. Inferred possible mutation events are represented by directed edges between nodes, with the arrowheads pointing to descendant spoligotypes. This specifies the cluster-graph [2].

Multiple inbound edges into a node are reduced to a single inbound edge. We use a heuristic that chooses a single inbound edge that has maximum weight. We define the weight *w *of an edge *e*_*AB *_in a cluster-graph from spoligotype *A *to its descendant *B *to be:

(7) $$w(e_{AB})=\frac{P(d(e_{AB}))\times n_{A}}{\sum_{i\in S}P(d(e_{iB}))\times n_{i}}$$

where *P*(·) is the model of deletion, *d*(·) is the deletion length represented by the edge, *n*_*i *_is the cluster size of spoligotype *i*, and *S *is the set of nodes that are possible parent nodes of spoligotype *B*. Ties in the maximum weight are broken arbitrarily. The resulting graph is what we refer to as a spoligoforest. Code for automatically constructing cluster-graphs and spoligoforests from a sample of tuberculosis spoligotypes was implemented using the visualization software GraphViz [19]. The method has been implemented on a web server and is publicly available at  (see [20] for details).

### Application of the method to tuberculosis spoligotypes

**Figure 2:**
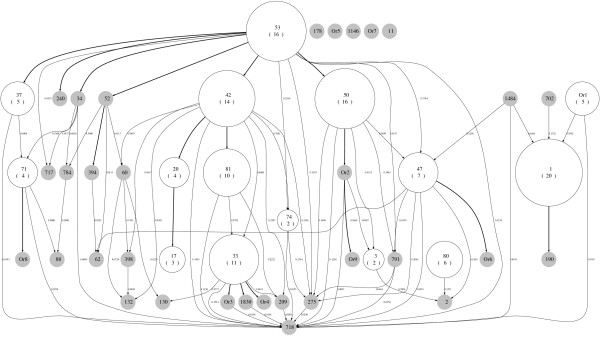
**Cluster-graph of Cuban data in Diaz et al**. [21]**with weighted edges**. Nodes are labelled with the ST identifier as indicated in SpolDB4 [22], with the cluster size enclosed in parentheses. Where the spoligotype does not appear in SpolDB4, it is called an orphan strain, hence labelled here 'Or' with a number. Sizes of nodes reflect the number of isolates sharing the spoligotype pattern associated with that node. Edges are labelled with corresponding weights that are computed as explained in the text. For example, ST 1 is inferred to have arisen either from ST 1484 (with weight 0.1666), ST 702 (with weight 0.2752) or Or1 (with weight 0.2809). The lengths of edges do not represent evolutionary distance.

**Figure 3:**
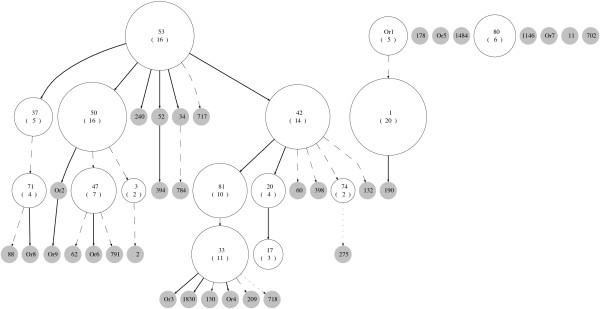
**The spoligoforest generated from the Cuban data in Diaz et al**. [21] Edges with weights less than 0.5 are drawn as dotted lines, those with weights greater than 0.5 but less than 1 are dashed, and those where no decision was required to be made are solid. For example, ST 1 is resolved to have mutated from Or1, and is drawn as a dashed line because it has a weight equal to 0.5582. As in the cluster-graph, the lengths of edges do not represent evolutionary distance.

We applied our new method for constructing forests to several data sets. To illustrate the method, we first use published data from a study on the transmission of tuberculosis in Cuba [21]. Isolates collected over a year were typed using both spoligotyping and IS*6110 *typing. One-hundred and fifty-seven isolates were classified into 47 spoligotype patterns. The clusters of isolates sharing the same spoligotype are nodes in the diagram, labelled using shared type (ST) numbers in SpolDB4 [22] wherever possible. When the spoligotype is not found in SpolDB4, we labelled it as 'Or' with a number (e.g. Or1). Orphan spoligotypes are unique alleles without an ST number [23]. Following the description in Tanaka and Francis [2], we constructed the cluster-graph for these data in a hierarchical layout as shown in Figure 2, with edges labelled with the weights computed using our selected model. The size of each node reflects the number of isolates in that node. The resulting graph is a complex network showing all possible relationships of spoligotypes under our assumptions about the spoligotype mutation processes. The Zipf model is used to calculate the weights of the edges, as given in Equation (7), of the cluster-graph. In this cluster-graph, there are 19 nodes with multiple inbound edges. The nodes are labelled according to the shared type (ST) identifiers used in SpolDB4 [22]. For example, ST 718 has 18 possible parents, while STs 47, 1, 62, 791, 2, 132 and 209, each has 3 possible parents. Of the 83 edges in the cluster-graph, 37 were chosen for the spoligoforest (see Figure 3). As with the cluster-graph, the nodes in the spoligoforest represent the number of isolates that share the same spoligotype pattern. If the weight of the edge is equal to 1, we draw a solid edge, if the weight is greater than or equal to 0.5 but less than 1, a dashed line, and if less than 0.5, a dotted line.

The spoligoforest consists of two trees (connected components) and eight disconnected nodes. The large tree has ST 53 at the root, suggesting that ST 53 is the oldest spoligotype in this tree. Seven spoligotypes are descended from ST 53, two of which have comparably large cluster sizes: ST 50 with 16 isolates and ST 42 with 14 isolates. These two spoligotypes form two distinct lineages diverging from ST 53. A comparison with the families in SpolDB4 [22] identified these two lineages to be the Haarlem and LAM (Latino-American and Mediterranean) families. ST 50 and its descendants ST 47 and ST 3 belong to the Haarlem family of strains, whereas ST 42 and its descendants STs 81, 20, 74, 33, and 17 are from the LAM family. ST 80, a disconnected node representing 6 isolates, is also of the Haarlem family. The separate smaller tree on the right includes ST 1 with 20 isolates. This is the spoligotype of the W-Beijing strain, known to be widely distributed around the world.

### Comparative analyses

In this section, we compare the spoligoforest to two other methods of visualisation, namely phylogenies and cladograms. We illustrate that using models with *AIC*_*c *_values close to that of the Zipf model has minimal effect on the edges of a spoligoforest.

The branches in a phylogeny show indirect relationships between isolates via implicit common ancestors, whereas the edges in the spoligoforest describe direct relationships among clusters of spoligotypes.

**Figure 4:**
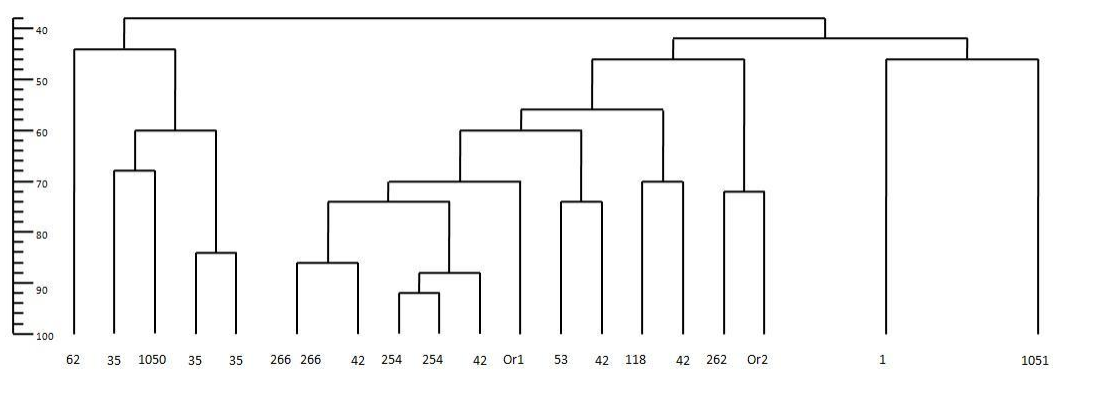
**IS6110-based phylogenetic tree from the Azerbaijan data in Pfyffer, et. al**. [24]. IS*6110*-based phylogenetic tree for data set in [24]. Each tip or leaf of the tree represents an isolate typed with both IS*6110 *and spoligotyping. The leaves are labelled with shared types (STs) from SpolDB4. Those spoligotype patterns not appearing in SpolDB4 (orphans) are labelled as Or1 and Or2. Forty-six isolates consisting of 25 different IS*6110 *profiles are all represented by ST 1.

**Figure 5:**
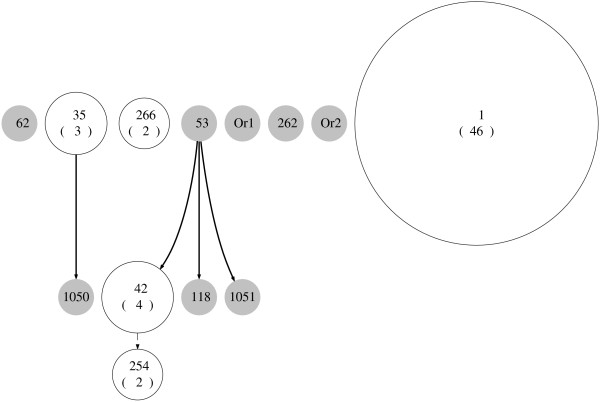
**Spoligoforest of the Azerbaijan data in Pfyffer, et. al**. [24]. Spoligoforest for data set in [24]. The labels of the nodes are the same as in Figure 4. ST 1, the W-Beijing spoligotype, is represented by the largest node. There are at least two clades revealed in the spoligoforest: the clade with ST 35 and ST 1050, and the clade with ST 53, 42, 254, 118 and 1051.

However, related spoligotypes in the spoligoforest are consistent with inferences on clustered isolates from a phylogeny. Figure 4 shows a phylogenetic tree based on IS*6110*-typing and Figure 5 is a spoligoforest using data from a prison in Azerbaijan [24]. The tree depicts genetic relatedness of isolates with each other based on similarities of IS*6110 *banding. The leaves of this tree have been renamed using STs (shared types from SpolDB4) of the spoligotypes, so that isolates sharing the same spoligotype may appear in different leaves of the tree. An inspection of the branch lengths in the phylogenetic tree indicate that ST 42 is most related to ST 254 (2 isolates of differing IS*6110 *bands.). The spoligoforest in Figure 5 is consistent with this observation: ST 42 is chosen as a parent for ST 254, with weight 0.7064 in the cluster-graph (not shown).

Furthermore, the spoligoforest shows that ST 42 is likely to have evolved from ST 53, which cannot be inferred from Figure 4. Direct links between spoligotypes are also seen in the spoligoforest, for instance the edge from ST 35 to ST 1050. In the phylogenetic tree, this relationship can be seen in the leftmost group with STs 35, 62 and 1050. Also, ST 1051 shown to be distant from the other types. It may be worth investigating whether ST 1051 is more related to the ST 53 group, as shown in the spoligoforest.

**Figure 6:**
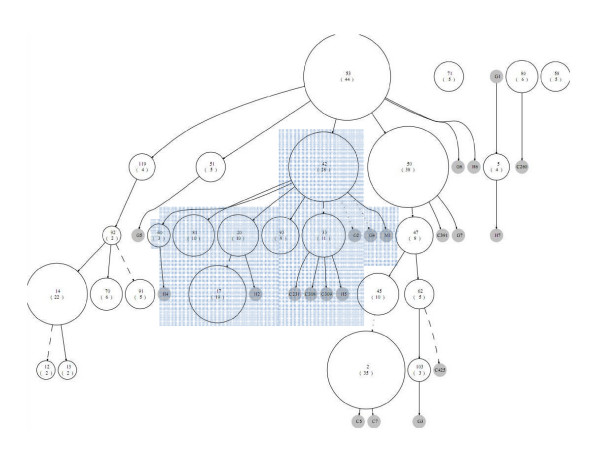
**Spoligoforest of Caribbean data in Duchene et al**. [25]. Spoligoforest for the Caribbean data set (from Cuba, Haiti and French Antilles) in [25]. The clustering of spoligotypes from the LAM group appears in the subtree with ST 42 at the top. This group is highlighted in the spoligoforest, and corresponds to clade 2-1 discussed in [25], which includes the LAM3 and LAM9 families.

A similar network-like technique of visualization to the spoligoforest is the cladogram in Figure 6 of [25]. The method of construction of the cladogram involves using information from nested clades and geographic location. The main difference between the cladogram and the spoligoforest is that the cladogram involves the introduction of intermediate steps between types, accounting for possibly unsampled spoligotypes. The spoligoforest for this data set is shown in Figure 6. The LAM3 and LAM9 groups identified in the cladogram are also evident as a subtree in the spoligoforest, with ST 42 at the top of this subtree (see highlighted region in Figure 6). The relationship of ST G4 with other spoligotypes is different in the two figures. In the spoligoforest, ST G4 is linked with a dotted line (computed weight of 0.2387 in cluster-graph, not shown) to ST 42. In the cladogram, however, ST G4 is related to ST 45 through a conjectured intermediate type. It may be interesting to assess whether ST G4 may be more related to the LAM3 and LAM9 groups than is shown by the cladogram.

**Figure 7:**
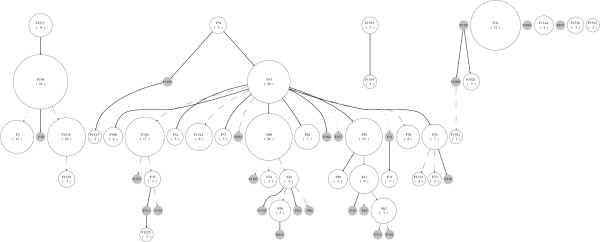
**Spoligoforest of Madagascar data in Ferdinand et al**. [26]**using Zipf model(Equation (6))**. Spoligoforest for the Madagascar data set in [26], generated using the selected Zipf model. The edges that are different from the spoligoforest in Figure 8 are F109→F1, F86→F1202, F47→F46 and F53→F237.

**Figure 8:**
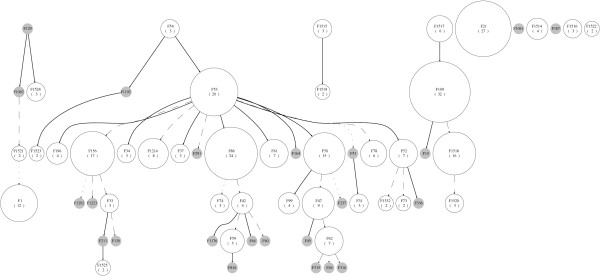
**Spoligoforest of Madagascar data in Ferdinand et al**. [26]**using alternate Logarithmic series model. (Equation (4))**. Spoligoforest for the Madagascar data set in [26], generated using the alternative Logarithmic series model. This graph differs from the spoligoforest in Figure 7 by the following edges: F1521→F1, F156→F1202, F62→F46 and F50→F237.

In order to assess the choice of model among the best four, we applied the method to several data sets using a range of possible models. This procedure has revealed that model selection has minimal impact on the edges of spoligoforests. We constructed the spoligoforests for six data sets, using the zeta, logarithmic series, geometric and empirical models. Table 7 shows the number of differing edges in spoligoforests constructed from these alternative models, relative to that constructed using the selected model (Zipf). Clearly, the Zipf and zeta models are similar, as the only difference between them is that the domain of the Zipf distribution is finite (see Table 3). The spoligoforest for the data set from Madagascar [26] using the selected Zipf model is shown in Figure 7. The spoligoforest using a logarithmic series model (Figure 8) for the same data set differs from Figure 7 by 4 edges, the highest number of edge differences among the data sets and models we tested.

**Table 7:** The number of edge differences between spoligoforests using alternate models as compared with selected model (Zipf)

Data set	Zeta	Logarithmic	Geometric	Empirical	No. of edges
Diaz et al. [21]	1	0	2	2	37
Duchene et al. [25]	0	3	3	2	42
Ferdinand et al. [26]	0	4*	0	0	50
Caws et al. [49]	0	2	2	1	44
Storla et al. [50]	0	0	2	2	42
Godreuil et al. [42]	0	0	1	1	22

*Spoligoforest shown in Figure 8.

## Discussion

This paper proposes a new method of visualizing the relationships among genotypes of tuberculosis by selecting a model of evolution of spoligotypes. The selected model is the Zipf model with parameter *p *for deletion length. We have made the spoligoforest application available in the spolTools website .

In this website, users can search through the repository of spoligotype data sets in spolTools as well as manipulate their own data sets. These data sets can be processed to construct spoligoforests.

A spoligoforest recovers a plausible history of transmission and mutation events. The area of each node is proportional to the number of isolates (cluster size); edges between nodes reflect evolutionary relationships between spoligotypes with arrowheads pointing to descendants. A single edge is chosen from multiple inbound edges using the deletion model, resulting in a forest – that is, a collection of acyclic graphs, or trees.

Information about the age of a spoligotype is contained in three aspects of a spoligoforest. First, if its node is large, the strain with that spoligotype may have been transmitted extensively over a long time. Second, a large number of descendants (outbound edges) suggests the strain has had a long period over which to generate mutations. Third, the location of a node also offers clues as to age: the closer it is to a root node, the older it is. For example, ST 1 in Figure 3 is a root and potentially the oldest spoligotype in this forest.

If a spoligotype node size is large yet located at a tip of the spoligoforest, this mixed signal may indicate that the strain with the spoligotype is transmitting faster than the other strains in the data set [17]. For example, ST 42 in Figure 3 has cluster size 14 and 6 outbound edges, whereas ST 81 with 10 isolates only has 1 outbound edge. ST 81 could therefore be an "emerging strain". Application of the analysis of Tanaka and Francis [17] did not, however, identify any rapidly spreading strains in this data set. In this case, therefore, there is no evidence for the presence of emerging strains. Note that the algorithm for choosing edges proposed in this study could be used to refine the method of Tanaka and Francis [17].

One way to improve the analysis of strain age and emergence would be to consider spoligotypes in conjunction with other molecular markers. For example, consider the same two spoligotypes discussed above (ST 42 and ST 81 from Figure 3). ST 42 has 12 different IS*6110 *profiles in the data set we used, while ST 81 has only one. This suggests once again that ST 81 may be associated with a higher transmission rate than ST 42. Further quantitative analysis would be needed to verify this point.

We note the limitations of our method. First, in choosing a single edge from multiple edges, we assumed that homoplasy (i.e., a spoligotype arising from more than one parent) does not occur. Because the number of spacers is finite and the deletion process is discrete, homoplasy may occasionally occur, but it is likely to be infrequent. The occurrence of homoplasy may have only a minor effect on graph-construction, producing a small number of cycles if such events could be properly identified. Second, we always choose one edge (parent) among possible inbound edges into a given spoligotype. It is conceivable, however, that the given spoligotype did not descend from any of the candidate parents. An improvement to the method would incorporate a procedure for not choosing any edges when appropriate. Third, as in any statistical analysis involving samples of data, there could be a bias in sampling. An overrepresentation of a spoligotype in a sampled data set can lead to biased selection of a parent node. Fourth, our methodology cannot be applied to markers such as Variable Numbers of Tandem Repeats (VNTR), which is commonly used to type various bacteria. The mutation process for VNTRs is better modeled using a stepwise mutation model rather than a deletion model.

Our method may, however, be suitable for markers based on loci of similar structure in some other bacteria. The direct repeat region of *M. tuberculosis *is among a family of repetitive genome sequences that are called Clustered Regularly Interspersed Short Palindromic Repeats (CRISPRs) found in many different species of bacteria and archaea [27-29]. Recently, CRISPR systems have received increased attention due to evidence that links these loci with the acquisition of resistance in bacteria to infection by phages [30]. Examples of these structures have been studied in *M. tuberculosis *[12,13], *Haloferax mediterranei *[31], *Methanocaldococcus jannaschii *[27,32], and *Yersinia pestis *[33].

The mechanisms that are believed to be involved in the evolution of CRISPR systems involve a frequent deletion of spacer-repeat motifs (thought to be necessary to prevent over-inflation of the CRISPR locus [29]) as well as the insertion of new spacers next to the leader sequence due to uptake of phage DNA [33].

Typing methods similar to spoligotyping for other bacterial isolates with CRISPRs are being developed. One such typing method (also called spoligotyping) has been applied to *Corynebacterium diphtheriae *strains, in which the location and structure of two CRISPR loci have been identified [34]. These loci consist of 27 spacers (the DRA with 21 spacers and the DRB with 6 spacers) in two different regions of the genome. The spoligotyping method used in this particular study is similar to the method used for *M. tuberculosis*. At present, there is yet to be an analysis of the evolution of these DR loci in *C. diphtheriae*. It has also been speculated that in some CRISPRs, new repeat motifs can appear, like those in *Yersinia pestis *[33]. Investigations into how these loci evolve may allow the development of methods similar to that described here.

As with other visualization methods, the groupings and relationships that are seen in the spoligoforest can be analysed along with the known clinical features of strains. Such analyses are valuable when an understanding of the history of transmission and mutation of strains is required.

## Conclusion

There is a lack of tools for visualizing relationships among tuberculosis isolates that employ a model describing evolution of a specific marker. Current understanding of the evolution of spoligotypes led us to a method for visualizing relationships of isolates within a sample. The methodology presented in this paper may be applied to loci that have the same structure as the DR region of *Mycobacterium tuberculosis*, and whose evolution involves the deletion of spacer-repeat motifs. The groupings and relationships that are seen in the spoligoforest can be analysed along with the clinical features of strains to understand the evolution of strains.

## Authors' contributions

JR implemented all the methods, performed the analytical and computational work and wrote the initial draft of the manuscript. MT, AF and JR designed the study and edited the manuscript.
